# Sex differences in psychotropic and analgesic drug use before and after initiating treatment with acetylcholinesterase inhibitors

**DOI:** 10.1371/journal.pone.0243804

**Published:** 2021-09-20

**Authors:** Anne Sverdrup Efjestad, Hege Ihle-Hansen, Vidar Hjellvik, Knut Engedal, Hege Salvesen Blix

**Affiliations:** 1 Hospital Pharmacy Ahus, Loerenskog, Hospital Pharmacy Enterprices, South Eastern Norway, Oslo, Norway; 2 Department of Neurology, Oslo University Hospital, Oslo, Norway; 3 University of Oslo, Oslo, Norway; 4 Norwegian Institute of Public Health, Oslo, Norway; 5 Norwegian National Advisory Unit on Ageing and Health, Vestfold County Hospital, Tønsberg, Norway; 6 Department of Geriatrics, Oslo University Hospital, Oslo, Norway; 7 Department of Pharmacy, University of Oslo, Oslo, Norway; Federico II University of Naples, ITALY

## Abstract

**Background/aims:**

The aim was to explore the impact of sex on prevalence, patterns and trends in the prescription of psychotropics and analgesics in users of acetylcholinesterase inhibitors (AChEIs), before and after AChEI initiation, compared to the general population.

**Methods:**

A prospective study applying data from the Norwegian Prescription Database (NorPD) in the period 2004–2016. Prescription of antidepressants, antipsychotics, analgesics including opioids, benzodiazepines and z-hypnotics in persistent AChEI users was studied in a follow-up period from four years before to two years after AChEI initiation in men and women of four age groups: 37–64, 65–72, 73–80 and 81–88 years.

**Results:**

Use of antidepressants, antipsychotics and weaker analgesics increased in both sexes during the follow-up period in 11.764 persistent AChEI users. Women with pre-dementia and dementia stages of AD showed a prescription pattern with more use of psychotropics and opioids than men, except for antipsychotics.

**Conclusion:**

Female sex showed to have a significant influence on the prescriptions of psychotropics and analgesics in AD patients in a pre-dementia and dementia stage. The exception is for antipsychotics, that men used more than women. The prescription pattern showed a higher extent of polypharmacy of psychotropics and/or opioids in women than in men. The total prescription pattern of analgesics could indicate an undertreatment of pain in pre-dementia and dementia stages, most pronounced in men.

## Introduction

Alzheimer´s dementia (AD) accounts for 60–80% of people with dementia with a greater proportion and severity of the disease in the higher age groups [1], resulting in 2/3 of patients with AD being women [2].

Approximately 90% of people with dementia develop at least one Behavioral and Psychiatric Symptom of Dementia (BPSD) like depression, agitation, psychosis, apathy or irritability over the course of the disease, symptoms becoming more common as the disease progresses [3]. BPSDs have been associated with sex for its biological characteristics, severity of dementia, and cultural background [4], often reflected in differences in prescription of medications [5]. Besides, disrupted sleep is common.

To stimulate cholinergic transmission, acetylcholinesterase inhibitors (AChEIs) are used in patients with mild to moderate degree of AD [6], with a hypothesized mechanism for improvement in cognition [7]. Some studies have suggested that these medications may alleviate BPSDs as well [8].

However, in most cases psychotropic drugs are used to treat BPSD. Antidepressants and anxiolytics are often used early in the course of AD, whereas antipsychotics are initiated later on [9, 10]. People with dementia use more psychotropics compared to the general population, despite the recommendations of a restrictive use of psychotropics in BPSD [11, 12]. Psychotropic polypharmacy, the use of two or more psychotropic drugs concomitantly, is generally associated with an increased risk of injurious falls, hospitalization and mortality and should be avoided in older persons with dementia [13].

Female sex has been shown to be associated with a higher likelihood of inappropriate drug use [14]. Further, women have an increased risk of polypharmacy [15], are more likely to have functional and cognitive disabilities due to aging and to live alone, known to be associated with depressed mood and over-utilization of psychotropic drugs [16]. Studies have shown that psychotropic drugs are used more common in hospitalised elderly women than in men [16]. However, antipsychotic drug use has been reported to be higher among men with AD and antidepressant drug use higher among women with AD [5, 17]. In Norway, older women living at home are reported to use more opioids and paracetamol than men [18], and female sex has been associated with an increased risk of drug related problems with opioids involved in nursing homes [19].

Thus, the aim was to explore the impact of sex on prevalence, patterns and trends in the prescription of drugs and co-medications of psychotropics and analgesics, as use of antidepressants, antipsychotics, benzodiazepines (BZDs), Z-hypnotics, opioids and weaker analgesics in users of AChEIs, compared to the general population.

## Materials and methods

### Data

Drug use data were collected from the Norwegian Prescription Database (NorPD) and include sex, age, year of death (when relevant) and information on prescribed drugs dispensed [10]. Drugs dispensed to patients in institutions are not included in the NorPD, making us unable to follow the patients’ drug use in nursing homes. NorPD contains a complete listing of all prescription drugs dispensed by pharmacies in Norway since 2004.

In this study, dispensed drugs represented consumed drugs. The drugs were classified according to the Anatomical Therapeutic Chemical (ATC) classification system version 2017 [20], obtaining date of dispensing, medicinal product name and formulation, ATC-code, number of defined daily doses (DDDs) and the number of tablets/capsules/plasters (allowing us to calculate treatment periods).

We had access to all persons being dispensed at least one prescription of AChEIs (ATC-code N06DA) and the yearly total number of users in the population by sex and one-year age groups (up to and including 89 years).

### Study population

We used the first prescription of an AChEI as a surrogate marker of the time of the AD diagnosis [11].

The study population consisted of all persistent (see below) AChEI users who initiated AChEI treatment between 1 January 2008 and 31 December 2013 at an age of 88 years or younger, who were alive two years after the year of AChEI treatment initiation, and who were registered in the NorPD the second year after AChEI initiation (not yet in nursing home). They were followed from 4 years (1460 days) before initiation to 2 years (729 days) after initiation. The age limit of 88 was set to make a comparison with the general population possible, for which data with one year age resolution was only available up to and including 89 years. The study population was stratified into four subgroups according to age at initiation. Due to the definition of early onset of dementia the young onset group include persons 37–64 years old. The last three groups were selected to reach equal age spans (65–72, 73–80 and 81–88 years old). Incident use was defined as being prescribed an AChEI drug after more than 365 successive days with no AChEI prescriptions. Some patients were repeated incident users (having several AChEI-free periods of more than 365 days). For each patient we started follow-up at the date of the prescription initiating the longest period with subsequent prescriptions less than 365 days apart (index date). The ‘longest period’ means the period with the largest number of prescriptions. Treatment length was estimated as the time from index date until the drug dispensed in the last prescription of the longest period was supposed consumed. The earliest possible index date was 1 January 2005. To allow for at least 1 year of follow-up we only included patients with index date before 1 January 2012

### Sex differences and use of drugs before and after initiation of AChEI

We studied sex differences in the prescriptions the last four years (365-day periods) before- and the first two years after AChEI initiation for the following drug groups: antidepressants (ATC code N06A excluding amitriptyline N06AA09, commonly prescribed for neuropathic pain), antipsychotics (ATC code N05A excluding prochlorperazine N05AB04, commonly prescribed for vertigo), opioids and weaker analgesics (ATC codes N02A and N02B), BZDs (ATC codes N05BA and N05CD) and Z-hypnotics (ATC code N05CF). The day of AChEI initiation was counted as the first day of the first year (365-day period).

Sex differences in prevalence of use of psychotropics and opioids, defined as proportion of users of antidepressants, antipsychotics, BZDs, Z-hypnotics, weaker analgesics and/or opioids, were studied in the cohort during the six years time interval. Use of a given drug class in a given 365-day period was defined as at least one prescription of a drug in the actual drug class in the actual period.

### Incident and persistent use of AChEI

Incident use was defined as being prescribed an AChEI drug after 365 successive days with no AChEI prescriptions. Since the treatment should, according to Guidelines, be evaluated 90–180 days after initiation, we considered due to possible delay, users who continued treatment 8 months (240 days) after initiation as persistent users [10]. Thus, an incident user was defined as persistent if any of the following was true [9]: i) a new prescription was given between day 210 and day 240 after initiation, ii) drugs for at least 210 days consumption were prescribed during the first 210 days from initiation, or iii) the last prescription before day 210 lasted to day 210.

### Sex differences and comparison with drug use in the general population

The use of e.g. antidepressants in the study population the first year after initiation of AChEI was compared to the use in the general population as follows: For each of the years 2008–2013, the age- and sex adjusted prevalence of antidepressant use in the general population was computed, using the age- and sex distribution of those initiating AChEI the actual year as reference. The over-all age- and sex adjusted prevalence in the general population was thereafter estimated as $\sum_{y=2008}^{2013}u_{y}/\sum_{y=2008}^{2013}N_{y}$ where *u*_*y*_ and *N*_*y*_ are the age-adjusted number of antidepressant users and the population in year *y*, respectively. When comparing the antidepressant use in AChEI initiators X years before/after initiation with the general population, the dispensing years and the age in the general population was shifted X years. As an example, antidepressant use in in the 81–88 year age group four years before initiation was compared to use in the 77–84 year old persons in 2004–2009 in the general population and antidepressant use in the same age group the second year after initiation was compared to use in the 82–89 year old in 2009–2014 in the general population. Age-adjusted prevalence ratios (PRs) for each sex were calculated by dividing the prevalence in the AChEI users by the age- adjusted prevalence in the general population.

### Statistical analysis

R version 3.1.0 [21] was applied for descriptive statistics; proportions with 95% confidence intervals (CIs) were computed for the study population. The CIs were computed using the ‘binom.confint’ function in the ‘binom’ package in R with method =“wilson”. Age adjusted prevalence rates were computed for the general population with the study population as reference, using the ‘ageadjust direct’ function in the ‘epitools’ package in R. The CIs for the general population are very narrow and hence not shown.

### Ethics

All the pharmacies in Norway register prescriptions electronically, and the information is sent in monthly reports to NorPD. The patient’s personal ID number and the prescribers ID number are replaced by a unique pseudonym by Statistics Norway. This makes it possible to link drug use to individuals without knowing their identity. Personal information is not disclosed from the NorPD. The database is governed by the national regulation of 17 October 2003 about the collection and processing of health data in the Norwegian Prescription Database. The NorPD generated pseudonymous files for research purposes, as regulated by Norwegian law for health registers [22], hence there was no demand of additional approval by the ethics committee.

## Results

**Fig 1:**
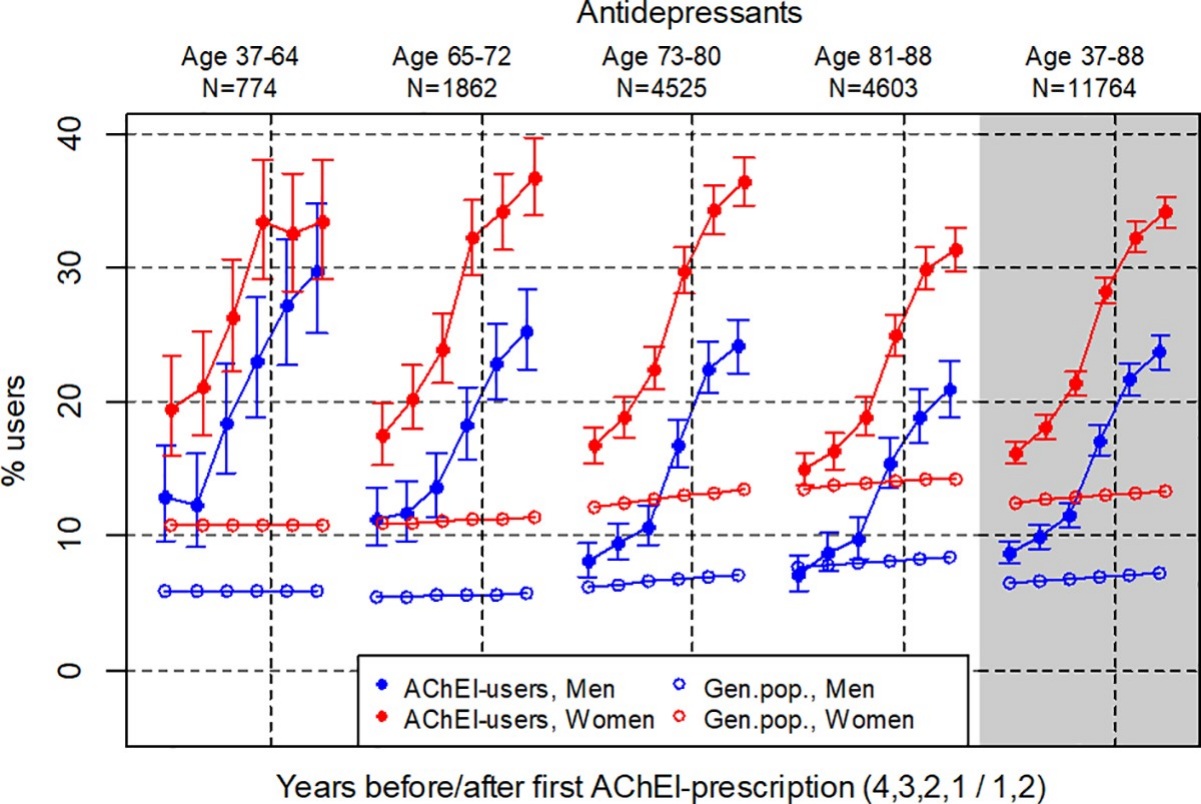
Bullets: Proportion of the study population who filled at least one prescription of antidepressants (ATC N06A except N06A09) the four years before AChEI initiation and the two years after with 95% confidence intervals. Circles: The corresponding age- adjusted proportion in the general population. Dashed vertical lines indicate AChEI initiation. The size of each age group in the study population is given on top.

**Fig 2:**
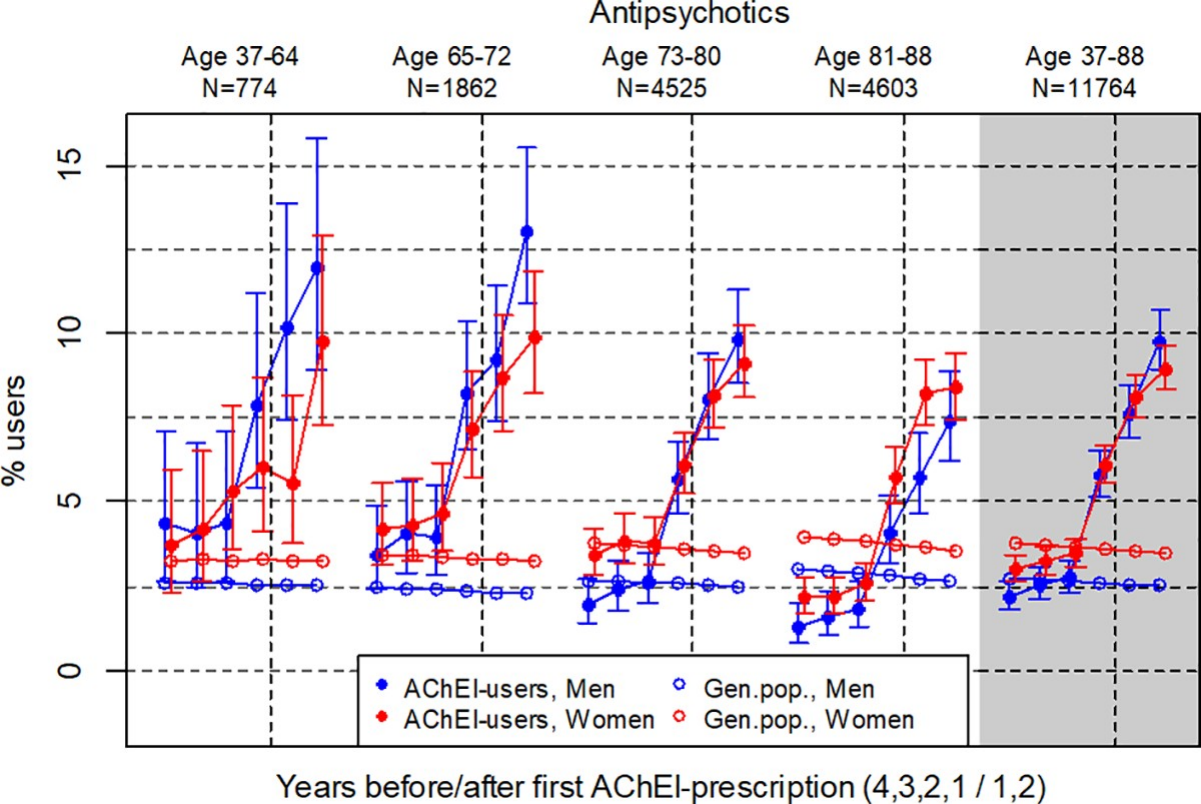
Bullets: Proportion of the study population who filled at least one prescription of antipsychotics (ATC N05A except N05AB04) the four years before AChEI initiation and the two years after with 95% confidence intervals. Circles: The corresponding age- adjusted proportion in the general population. Dashed vertical lines indicate AChEI initiation. The size of each age group in the study population is given on top.

**Fig 3:**
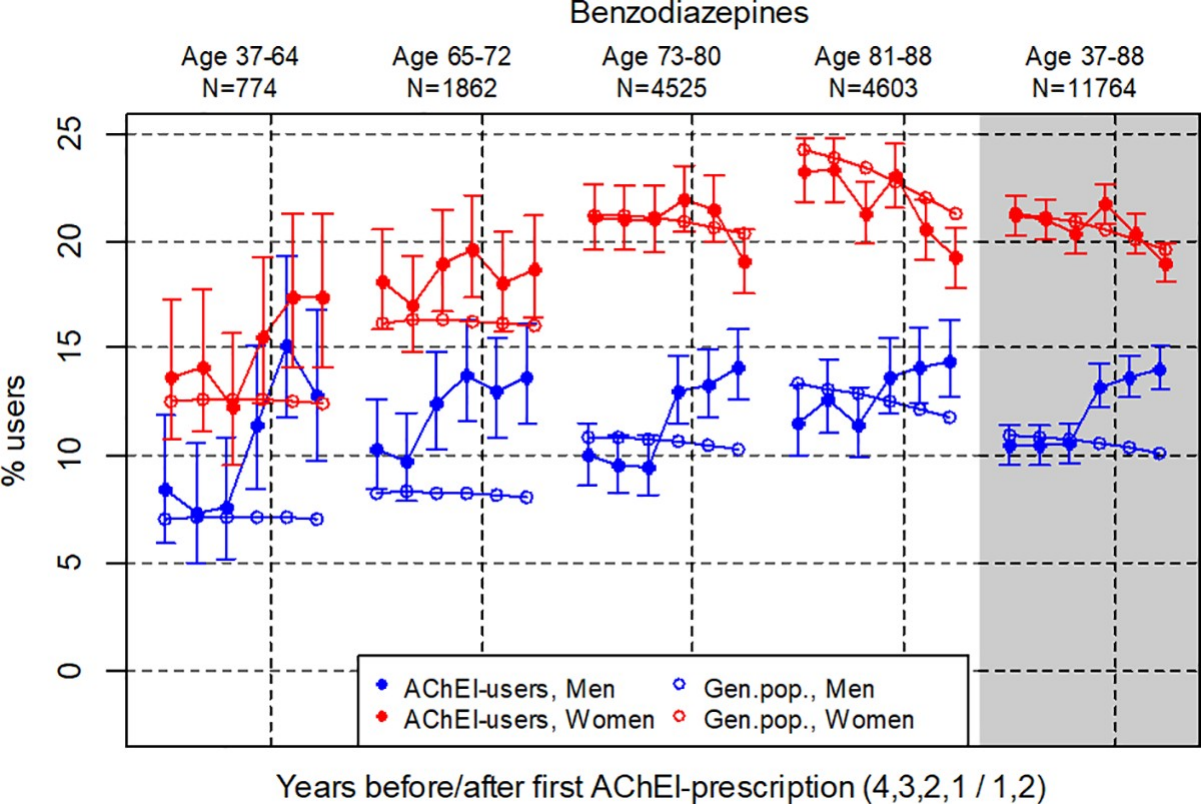
Bullets: Proportion of the study population who filled at least one prescription of benzodiazepines (ATC N05BA or N05CD) the four years before AChEI initiation and the two years after with 95% confidence intervals. Circles: The corresponding age- adjusted proportion in the general population. Dashed vertical lines indicate AChEI initiation. The size of each age group in the study population is given on top.

**Fig 4:**
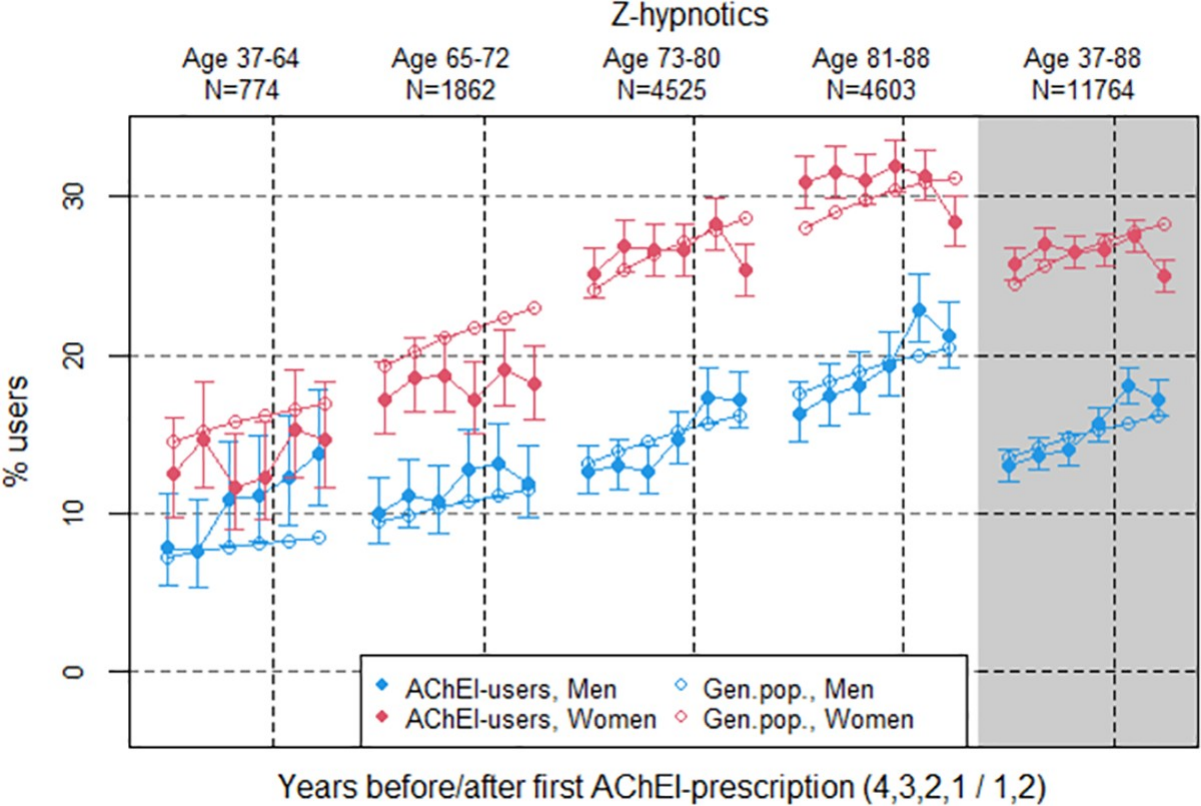
Bullets: Proportion of the study population who filled at least one prescription of Z-hypnotics (ATC N05CF) the four years before AChEI initiation and the two years after with 95% confidence intervals. Circles: The corresponding age- adjusted proportion in the general population. Dashed vertical lines indicate AChEI initiation. The size of each age group in the study population is given on top.

**Fig 5:**
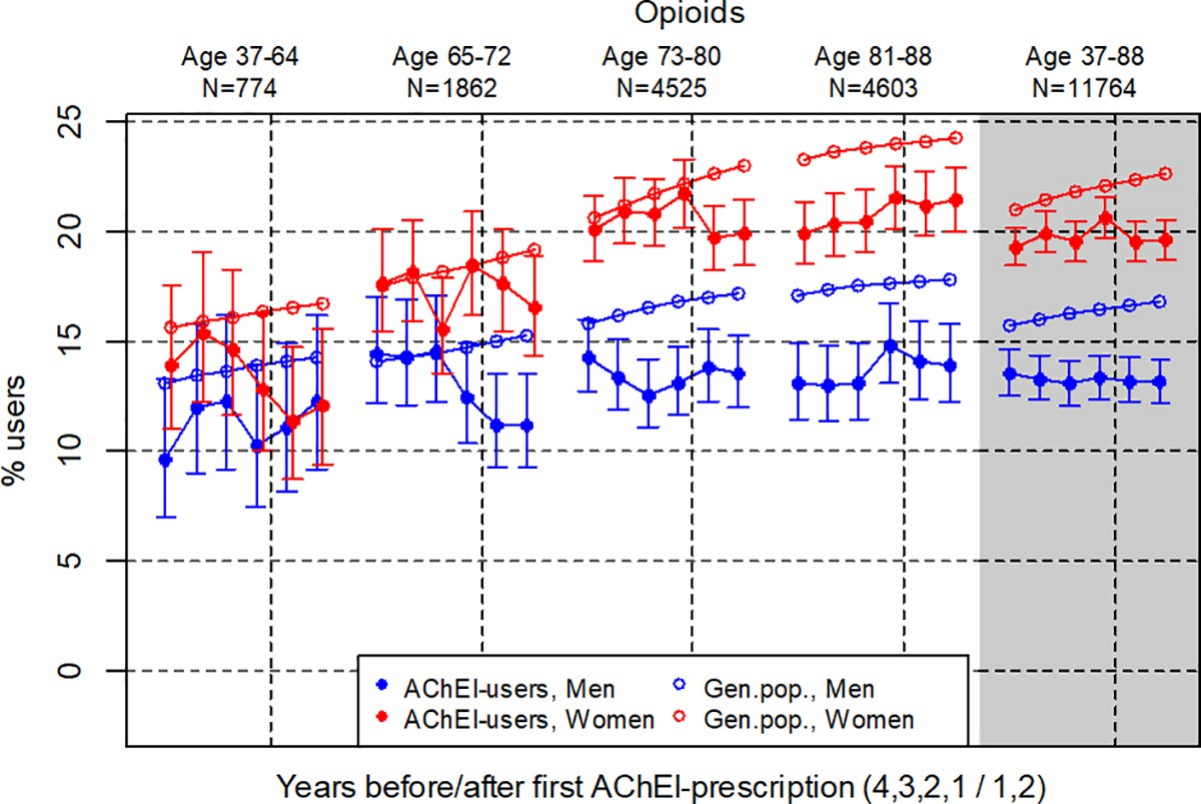
Bullets: Proportion of the study population who filled at least one prescription of opioids (ATC N02A) the four years before AChEI initiation and the two years after with 95% confidence intervals. Circles: The corresponding age- adjusted proportion in the general population. Dashed vertical lines indicate AChEI initiation. The size of each age group in the study population is given on top.

**Fig 6:**
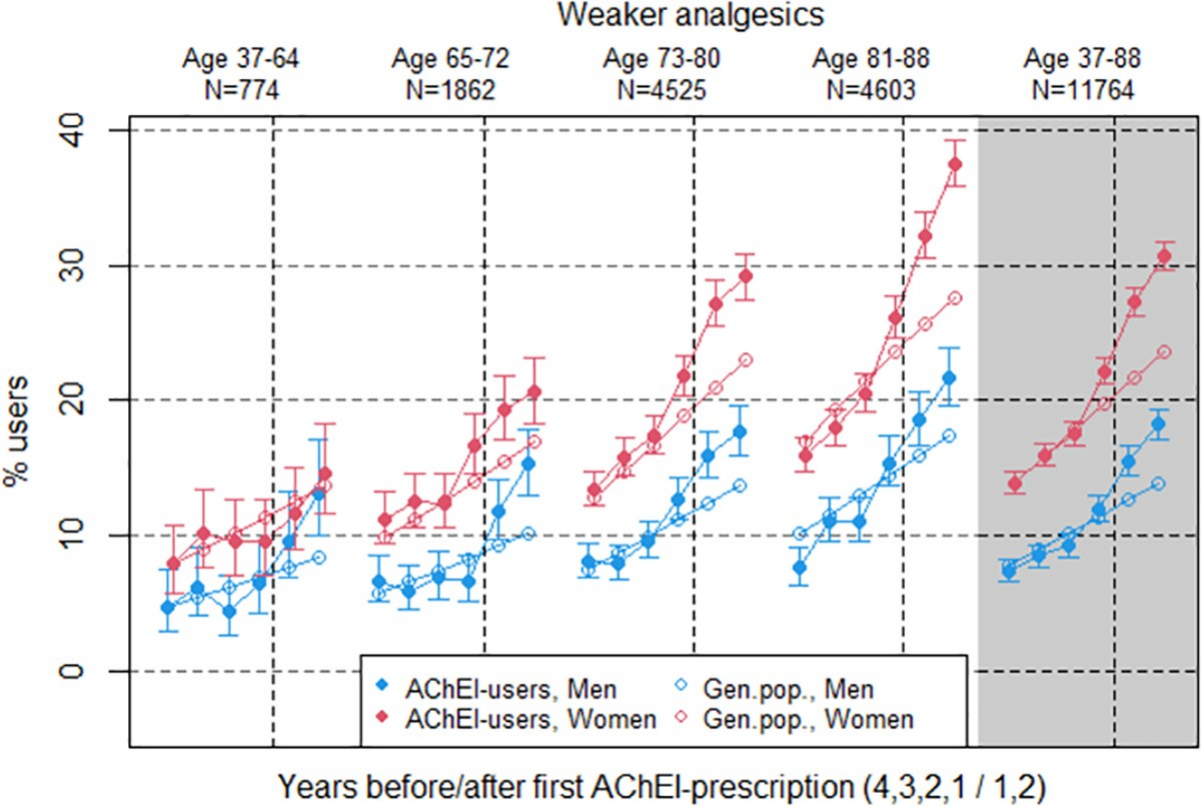
Bullets: Proportion of the study population who filled at least one prescription of weaker analgesics (ATC codes N02A and N02B) the four years before AChEI initiation and the two years after with 95% confidence intervals. Circles: The corresponding age- adjusted proportion in the general population. Dashed vertical lines indicate AChEI initiation. The size of each age group in the study population is given on top.

The study population consisted of 11.764 persistent AChEI users aged 37–88 years who initiated treatment between 1 January 2008 and 31 December 2013. The percentage of women in the four age groups was 56%, 56%, 61%, and 68%, respectively, and 63% in the full study population. The proportion of the study population receiving at least one prescription of antidepressants, antipsychotics BZDs, Z-hypnotics, opioids and weaker analgesics the four years before initiation of AChEI and the two years after, are shown in Figs 1–6, respectively, together with the age-adjusted proportion of the general population receiving the same drugs.

We found no differences between age-groups related to prescription of the various AChEIs. As donepezil was the first AChEI on the market between 69% (youngest age group) and 72% (oldest age group) used this drug.

### Prevalences of use in the general population

Women of the general population showed a higher prevalence of use of antidepressants, antipsychotics, benzodiazepines, Z-hypnotics, opioids and weaker analgesics than men. A notable age-related increase in use of these drug groups was observed, especially in women, except for antipsychotics. Within each age-group in Figs 1–6, the change in prevalence over the 6 years covered can be attributed both to a 6 year increase in age and a 6 year increase in calendar year. The change was small for most of the drug groups: In the 37–88 year age group the prevalence for *antidepressants* increased from 6.5% to 7.2% in men and from 12.5% to 13.3% in women; for *antipsychotics* it decreased from 2.7% to 2.5% in men and from 3.8% to 3.5% in women; for *BZDs* it decreased from 10.6% to 9.9% in men and from 20.9% to 19.3% in women; for *Z-hypnotics* it increased from 13.4% to 16.1% in men and from 24.6% to 28.3% in women; for *opioids* it increased from 15.7% to 16.8% in men and from 21.0% to 22.6% in women. For *weaker analgetics*, however, the prevalence almost doubled over the 6 years from 7.8% to 13.8% in men and from 13.9% to 23.6% in women. For all of the drug groups except BZDs, the pattern of change was the same in all age groups, but for BZDs the prevalence was stable in the youngest age group (at 6.8% for men and 12.1% for women) but markedly decreasing in the oldest age group (from 13.1% to 11.5% in men and from 23.9% to 21.0% in women). Due to the large sample size, the width of the 95% confidence intervals are less than 0.16 in all cases, and thus all of the changes are statistically significant, but none are large enough to be of any clinical significance.

### Prevalences of use in the study population

Women of the study population showed a higher prevalence of use of antidepressants, BZDs, z-hypnotics, opioids and weaker analgesics than men. A notable age-related increase in use of these drug groups was observed, except for antidepressants and antipsychotics.

From four to one year before initiation of AChEI the prevalence of use in the study population overall (age 37–88) compared to the general population was higher for antidepressants, lower for antipsychotics and opioids, and similar for BZDs, Z-hypnotics and weaker analgesics. From one year before to two years after initiation, however, the prevalence in the study population was higher than in the general population also for antipsychotics, weaker analgesics, and BZDs (men only), and lower only for opioids. There were some age group specific exceptions to this pattern, though, se details for the different drug groups below.

### Antidepressants

The use of antidepressants strongly increased in both genders over the six years interval and was higher in women than in men in all age groups (Fig 1). The use in women increased from 16.2% (95% CI: 15.4%-17.1%) the fourth year before initiation to 34.1% (33.0%-35.2%) the second year after. The corresponding increase in men was from 8.8% (95% CI: 8.0%-9.6%) to 23.7% (22.5%-25.0%). Comparing with the general population, the prevalence ratio (PR) increased from 1.3 to 3.3 over the six years in men and from 1.3 to 2.6 in women. In the youngest age group, the prevalence four year before initiation was about twice as high in the study population as in the general population, whereas in the oldest age group it was about the same in the two populations. In all age groups the prevalence increases strongly the years before AChEI initiation, but in the youngest age group there is no significant further increase after initiation.

### Antipsychotics

The prevalence of the use of antipsychotics strongly increased in the study population overall (age 37–88) in both sexes from two years prior to the dementia diagnosis and during the two years following initiation of AChEIs (Fig 2). The use in women increased from 3.0% (95% CI: 2.7%-3.4%) the fourth year before initiation to 9.0% (95% CI 8.3%-9.6%) the second year after, respectively. The corresponding increase in men was from 2.2% (95% CI: 1.8%-2.7%) to 9.8% (8.9%-10.7%). Comparing with the general population, the PR in men increased from 0.8 the fourth year before initiation via 1.0 the second year before to 3.9 the second year after initiation, and in women from 0.8 via 1.0 to 2.6. In the youngest age group, the prevalence was higher in the study population than in the general population all six years (although the difference was not statistically significant the first years), whereas in the oldest age group the prevalence in the study population four years before initiation was only about half the prevalence of the general population. Two years before initiation it was still significantly lower (at 2.6% in women and 1.8% in men), but then increased markedly the year before initialization to 5.7% and 4.1% in women and men, respectively, and further to 8.4% and 7.4% the second year after initialization

### BZDs

The prevalence of the use of BZDs was higher in women than in men during the study period (Fig 3), however, continued to rise following AChEI initiation in men, but decreased in women the years after AChEI initiation, compared to the year before. Over the six years the use in women decreased from 21.1 (95% CI 20.2%-22.1%) to 19.0% (18.1%-19.9%), whereas in men it increased from 10.4% (9.6%-11.4%) to 14.0% (13.0%-15.1%). In men, most of the increase occurred from the second to the first year before initiation. Compared to the general population, the use of BZDs was highest in the youngest age group the two years after AChEI initiation (average PR of 2.1 for men and 1.4 for women). The PR in the 37–88 age group was stable at 1.0 for women and increased from 1.0 to 1.4 for men.

### Z-hypnotics

The prevalence of the use of z-hypnotics was higher in women than in men in the study population during the study period, however, decreased the second year after AChEI initiation, more strongly in women than in men (Fig 4). The use in women decreased from 25.8% (95% CI 24.8%-26.8%) to 25.0% (24.0%-26.0%), whereas in men it increased from 13.0% (12.0%-14.0%) to 17.2% (16.1%-18.4%). Compared to the general population the PR was close to 1 for both genders.

### Opioids

The prevalence of the use of opioids was lower in men than in women in the study population during the study period (Fig 5), and quite stable for both sexes. The use in women increased from 19.3% (95% CI 18.4%-20.2%) to 19.6% (18.7%-20.5%), whereas in men it decreased from 13.5% (12.5%-14.6%) to 13.1% (12.1%-14.1%). Compared to the general population the PR was stable at 0.9 over the six years for women and decreased from 0.9 to 0.8 for men. The largest difference between the study population and the general population was observed in the oldest age group.

### Weaker analgesics

The prevalence of the use of drugs of weaker analgesics, mainly consisting of paracetamol, was increasing in the study population during the study period and was higher in women than in men (Fig 6). The first three years it was very close to the prevalence in the general population, but increased more rapidly the last three years, for both women and men. The use in women increased from 13.9% (95% CI 13.1%-14.7%) to 30.6% (29.6%-31.7%) over the six years, and in men from 7.4% (6.7%-8.2%) to 18.2% (17.1%-19.4%). Compared to the general population the PR in the 37–88 year age group increased from 1.0 to 1.3 in women and from 0.9 to 1.3 in men, with all of the increase taking place the last three years. The pattern was similar in the four age groups, except for the youngest group where the increase over the last three years was weaker.

### Psychotropic polypharmacy and opioids

**Fig 7:**
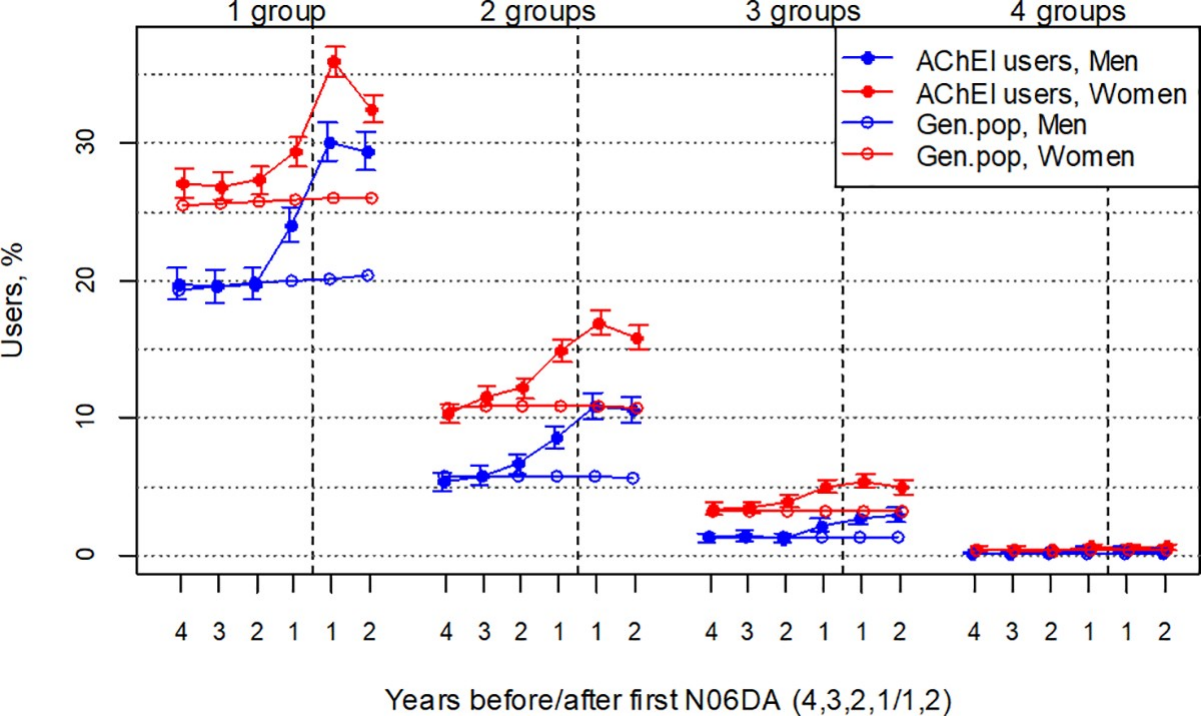
Bullets: Proportion of the study population with concomitant use of exactly 1, 2, 3 or 4 of the drug groups antidepressants, antipsychotics, BZDs and opioids in the 4 years before AChEI initiation and the 2 years after initiation, with 95% confidence intervals. Circles: The corresponding age-adjusted proportion in the general population. Dashed vertical lines indicate AChEI initiation.

**Fig 8:**
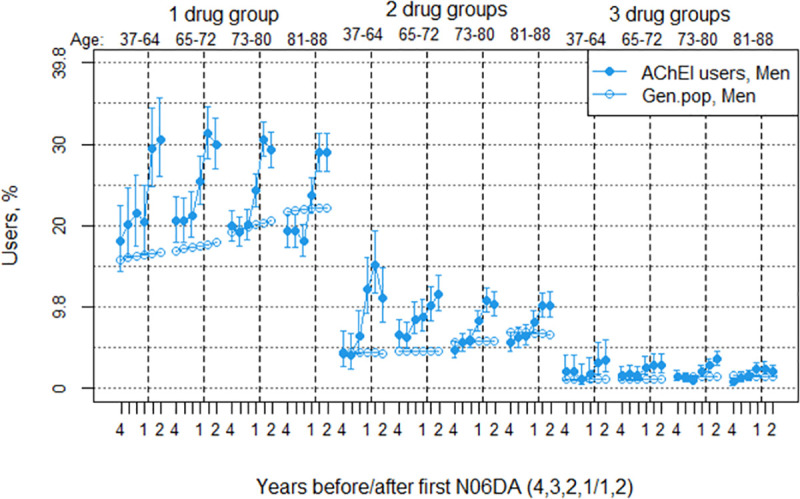
Bullets: Proportion of the study population of men with concomitant use of exactly 1, 2 or 3 of the drug groups antidepressants, antipsychotics, BZDs and opioids in the 4 years before AChEI initiation and the 2 years after initiation, with 95% confidence intervals. Circles: The corresponding age-adjusted proportion in the general population. Dashed vertical lines indicate AChEI initiation. The size of each age group in the study population is given on top.

**Fig 9:**
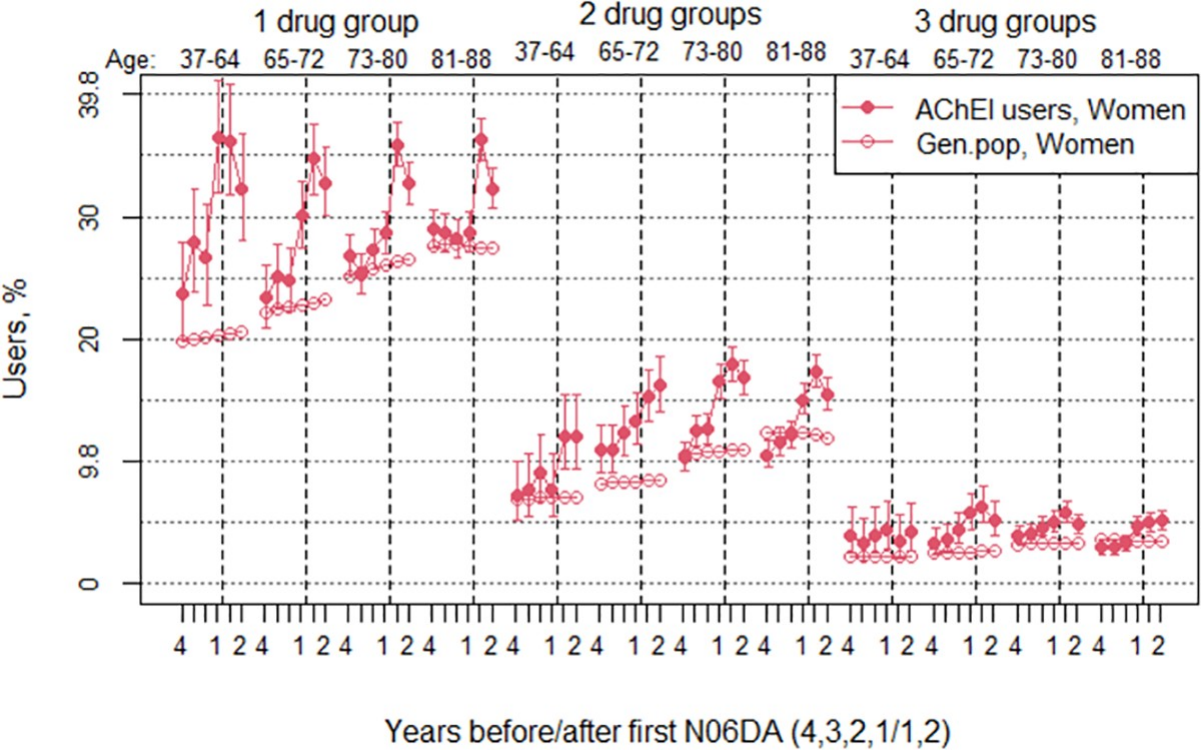
Bullets: Proportion of the study population of women with concomitant use of exactly 1,2 or 3 of the drug groups antidepressants, antipsychotics, BZDs and opioids in the 4 years before AChEI initiation and the 2 years after initiation, with 95% confidence intervals. Circles: The corresponding age-adjusted proportion in the general population. Dashed vertical lines indicate AChEI initiation. The size of each age group in the study population is given on top.

The proportion of the study population with use of 1 and 2 of the drug groups increased in both sexes the years before AChEI initiation and the first year after, and then flattened out or slightly decreased the second year after in both sexes. The proportion of the study population with concomitant use of 3 and 4 drug groups was low in both sexes (Fig 7). The proportion of women in the study population with use of one drug group increased from 27.1% (95% CI 26.1%-28.1%) four years before AChEI initiation to 35.9% (34.8%-37.0%) the first year after. The corresponding increase in the general population was from 25.5% to 26.0% (Fig 7). The proportion of women with two drug groups increased from 10.3% (9.6%-11.0%) to 17.0 (16.1%-17.8%) in the study population and from 10.7% to 10.8% in the general population. The proportion of men in the study population with use of one drug group increased from 19.7% (18.6%-20.9%) to 30.1% (28.7%-31.5%). The corresponding increase in the general population was from 19.3% to 20.2%. The proportion of men with two drug groups increased from 5.3% (4.7%-6.0%) to 10.8% (9.9%-11.8%) in the study population, whereas it was stable at 5.7% in the general population. The PR between the study population and the general population was in general highest for the young age groups, both for use of 1, 2 and 3 drug groups, in particular so for the years before AChEI initiation (Figs 8 and 9). The proportions with 4 groups were very low and are not shown.

### Summary

We observed a marked increase in the use of antidepressants, antipsychotics and weaker analgesics in both sexes in AChEI users the last two years prior to AChEI initiation, and the use of these drugs continued to rise the first two years after initiation. However, a decreasing prevalence in the use of BZDs and Z-hypnotics following AChEI initiation in women was observed.

Women of the study population had a higher prevalence of use of the studied drug classes compared to the general population except for opioids and Z-hypnotics, and a higher prevalence of use of the studied drug classes than men except for antipsychotics. The prevalence of opioids was lowest in the lowest age group in both sexes.

The highest prevalence rates of antidepressants and antipsychotics were observed in the lower age groups in both sexes, and we observed a tended high use of antipsychotics in men in the lower age groups.

The proportion of the study population with use of a drug in either one or two of the drug groups of antidepressants, antipsychotics, BZDs and/or opioids increased in both sexes the years before AChEI initiation and decreased slightly from one year after in both sexes, most pronounced in women (Fig 7). As much as 17% of the women and 11% of the men in the study population were prescribed drugs in two of the drug groups the first year after AChEI initiation. Corresponding numbers for one drug group was 36% and 30%, and for three drug groups 5 and 3%, respectively.

## Discussion

Female sex showed to have a significant influence on the prescriptions of psychotropics and analgesics in AD patients in a pre-dementia and dementia stage. The exception is antipsychotics that men used more than women. The prescription pattern showed a higher extent of polypharmacy of psychotropics and/or opioids in women than in men.

The increase in prevalence in the use of antidepressants, antipsychotics and weaker analgesics in both sexes in AChEI users with pre-dementia and dementia conditions probably mirrors that BPSD increases in intensity and in symptomatology with increasing severity of AD, without achieving the desired effect of the initially initiated drug treatment. Thus, the change in prevalence of use of these drug groups appears to take place independently of AChEIs. The higher use of psychotropics in women in the study population indicates that women with AD experience BPSD more frequently than men, as shown in other studies [5]. Older women are likely to have more physical and mental co-morbidities than men and this may in part explain differences in psychotropic medication use between sexes [5]. Severe physical disorders in people with dementia increase the probability of delirium, which often is treated with psychotropic drug.

The proportion of the study population with concomitant use of exactly one, and two of the drug groups of antidepressants, antipsychotics, BZDs and/or opioids decreased from one year following AChEI initiation in women and flattened out in men.

We observed a decreasing prevalence in the use of BZDs and Z-hypnotics in women and in z-hypnotics in men. This may reflect a precaution to reduce the extent of co-medications in people with an AD diagnosis, especially in women

The high prevalence of use of antidepressants the years before initiation of AChEIs indicates that depression could be a preclinical symptom of AD in both sexes, and possibly that women generally express depressive thoughts better and more often than men. Also, women have been reported to experience depressive symptoms more frequently than men [5], to be more vulnerable to depression than male patients in the mild dementia group [23], to be overtreated with antidepressants and to be prescribed SSRIs more often than men [24]. The increase in prevalence of use might have started more than four years before initiation of treatment with AChEI and is not necessarily related to the initiation. Antidepressants have been reported to be prescribed for other indications than depression in 50% of the cases [25] such as pain and insomnia [26]. According to the national Norwegian dementia guidelines, a SSRI drug should only be offered as additional treatment to patients, when appropriate environmental psychological and/or psychotherapeutic measures have been attempted without achieving the desired effect [11]. The high use in AD patients, especially in women, compared to the general population may be inappropriate, but could also indicate that other treatment strategies have either failed or not been offered. This is worrisome in light of potential severe adverse effects antidepressants may induce [8, 27].

The effect of antipsychotics in BPSD is modest, however, their risk profile is extensive, and a restricted recommended use in Norway for the treatment of BPSD is described in the national dementia guidelines [11]. Despite the recommendation of the national guidelines, the use of antipsychotics in both sexes showed an increasing and high use during the study period. The higher use in men could be explained by the observed differences of BPSD between sexes. Men with dementia are often described as being more physically aggressive compared to women, which may result in more use of antipsychotics in men [5, 17]. The tended high use of antipsychotics in men in the study population in the lower age groups indicates this kind of differences in BPSD. Associations have been found between pain and depression, and depression plays an important role in development of agitation [28]. The use of antipsychotics may therefore be related to pain and depression as agitation often is treated with antipsychotics. Anxiety disorders are the most common psychiatric diseases, and women are twofold more likely than men to develop anxiety at disorder level during lifetime [16], and this is also the case for people with dementia, with anxiety being highly prevalent [29]. Besides, dependency-producing properties of anxiolytics are more pronounced in women than in men [16]. The use of BZDs have been connected to the risk of development of AD, especially the short-acting agents [30], however, another possible explanation is that insomnia could be a prodromal symptom of AD [31].

The use of BZDs and Z-hypnotics was increasing by age, for BZDs especially in women, The high prescription pattern of BZDs for women in particular in the higher age groups, is therefore of concern.

The use of anxiolytics among women has been associated with depressive disorders. This may be due to women tending to have pronounced anxiety symptoms of depression [32], and may suffer from a mixed depression/anxiety condition and that these symptoms are not recognized as early symptoms of AD. Another explanation could be that women in general use more anxiolytics compared to men. We observed an increasing prevalence in the use of BZDs in men from two years before the AD diagnosis, which could indicate that men become more noisy or sleepless at night during the course of the disease.

Women have a much higher prevalence of many pain disorders than men, and sex differences in the use of analgesics probably mirror the higher prevalence of chronic pain in women [33]. The lower use of opioids in men compared to women could reflect an undertreatment of pain, partially explained by the challenges associated with assessing pain, especially in patients with severe dementia, and probably not compensated for by increased use of weaker analgesics, which was strongly increasing in women in the oldest age group. One can ask whether the low use of opioids was related to the tended high use of antipsychotics in men in the lower age groups.

It should be noted that data in the figures of the study population overall (age 37–88) mirror data of the two oldest groups more than the two lower age groups, as the number of subjects in the two oldest groups represent nearly 75% of all subjects in the study population. Thus, the data for the study population overall should be interpreted with that in mind.

Psychotropic polypharmacy is reported to be frequent in AD patients [34], especially in women [35]. Demented women more often concurrently use drugs known to impair cognition [36] compared to men, with a higher risk of adverse events and death [37]. The increased risk may be driven by combinations with BZDs [37]. Other studies show that about 20% of patients with dementia living at home have been reported to be prescribed two or more psychotropic drugs [34] and similar results were found in the study population. As much as 36% of the women and 30% of the men in the study population were prescribed psychotropics and/or opioids one year following AChEI initiation. Although the prevalence of using psychotropics and/or opioids tended to decrease following AChEI initiation, the prevalence rates were still high (17% vs 11%). An important question is whether psychotropic polypharmacy is useful in patients with dementia, given the known risks and potential drug interactions [34], which is of concern especially in women of the study population. Potential pharmacodynamics and/or pharmacokinetic interactions among different drugs may contribute as a potential explanation underlying the decreased occurrence of polypharmacy the second year after initiation of treatment of AD patients with AChEIs. In addition, the findings in this study may not necessarily be generalized to other populations, especially since our data is not based on diagnoses, function or comorbidities.

### Strengths and limitations

Data from the NorPD gives us a unique opportunity to study drug use patterns, highlighting changes over time in the selected drug groups. The large sample size and the long study period is a strength in our study. A limitation is that patients in institutions are not included in NorPD and therefore not included in the present study, hence the patients with the most severe symptoms of AD is probably not included in our study. The NorPD does not include data on the use of over-the-counter drugs and herbal drugs which may lead to an underestimation of weak analgesics in our study as paracetamol in small pack sizes are available as over-the-counter drugs. AChEIs are mainly prescribed for treatment of AD, however, we did not have information with regard to clinical rationale for prescriptions and indications. Purchased drugs were used as a surrogate for consumed drugs and may cause overestimation. However, medicine adherence in AChEI users is expected to be good, as caregivers usually are responsible for drug management for patients with dementia. We did not study dosage differences between men and women, which could be of relevance due to pharmacokinetic differences between the sexes. Concerning prevalence of psychotropic polypharmacy and opioids, we only record ATC classes and not number of substances. This probably leads to an underestimation of prevalence of polypharmacy. We do not know the duration of symptoms before the diagnosis, and generally, the described increases in prevalence of use of psychotropic drugs might have been started more than four years before AChEI initiation. We also assume younger people to seek medical attention earlier than older people, however, early onset dementia is not always suspected and probably rather interpreted as depression.

## Conclusion

In conclusion, the increasing prescription pattern in the use of antidepressants, antipsychotics and weaker analgesics in both sexes during the study period probably mirrors that BPSD, sleep disturbances and pain increase in intensity or in symptomatology with increasing severity of AD, without achieving the desired effect of the initially initiated drug treatment. Female sex showed to have a significant influence on psychotropic prescribing. Women with pre-dementia and dementia stages of AD showed a prescription pattern with more polypharmacy of psychotropics and opioids than men, except for antipsychotics. The total prescription pattern of analgesics could indicate an undertreatment of pain in pre-dementia and dementia stages, most pronounced in men. Sex needs to be taken into consideration in clinical practice in treatment of pre-dementia and dementia conditions, to improve patient outcomes.

## Supporting information

S1 Data Aggregated data supporting Figs 1–9. (XLSX)
